# Pose and Focal Length Estimation Using Two Vanishing Points with Known Camera Position

**DOI:** 10.3390/s23073694

**Published:** 2023-04-03

**Authors:** Kai Guo, Rui Cao, Ye Tian, Binyuan Ji, Xuefeng Dong, Xuyang Li

**Affiliations:** Northwest Institute of Nuclear Technology, Xi’an 710024, China; ty_2122022@163.com (Y.T.); jibinyuan163@163.com (B.J.); dongxuefeng15@163.com (X.D.); lixuyang0827@163.com (X.L.)

**Keywords:** pose estimation, vanishing point, focal length, camera position, unit direction vector

## Abstract

This paper proposes a new pose and focal length estimation method using two vanishing points and a known camera position. A vanishing point can determine the unit direction vector of the corresponding parallel lines in the camera frame, and as input, the unit direction vector of the corresponding parallel lines in the world frame is also known. Hence, the two units of direction vectors in camera and world frames, respectively, can be transformed into each other only through the rotation matrix that contains all the information of the camera pose. Then, two transformations can be obtained because there are two vanishing points. The two transformations of the unit direction vectors can be regarded as transformations of 3D points whose coordinates are the values of the corresponding unit direction vectors. The key point in this paper is that our problem with vanishing points is converted to rigid body transformation with 3D–3D point correspondences, which is the usual form in the PnP (perspective-n-point) problem. Additionally, this point simplifies our problem of pose estimation. In addition, in the camera frame, the camera position and two vanishing points can form two lines, respectively, and the angle between the two lines is equal to the angle between the corresponding two sets of parallel lines in the world frame. When using this geometric constraint, the focal length can be estimated quickly. The solutions of pose and focal length are both unique. The experiments show that our proposed method has good performances in numerical stability, noise sensitivity and computational speed with synthetic data and real scenarios and also has strong robustness to camera position noise.

## 1. Introduction

The camera pose estimation, using accurate inputs, is an old but still widely studied topic. The accurate inputs are mainly points, lines and planes. If the relative pose needs to be estimated, the 2D–2D point or line correspondences are used, and many algorithms have been proposed [1,2,3,4,5]. If the absolute pose needs to be estimated, the 2D–3D point or line correspondences are used, and the corresponding methods are called PnP (perspective-n-point) solvers [6,7,8,9] and PnL (perspective-n-line) solvers [10,11,12,13]. In addition, there is a category of methods between the point-based and line-based correspondences, such as the pose estimation methods using vanishing points [14,15]. The 3D parallel lines in space will intersect at a point in the image plane called the vanishing point. When these methods estimate the pose, the 3D parallel lines and 2D vanishing points will be used. The method proposed in this paper is based on the vanishing points to estimate the focal length and pose. It also needs accurate inputs, which are similar to the PnP and PnL solvers. Hence, we briefly introduce the two categories of methods as follows.

When there is no other prior knowledge, the minimum point set for solving the PnP problem is three, and the corresponding methods are called P3P (perspective-three-point) solvers [16,17,18,19], which have a maximum of four solutions, showing the phenomenon of multiple solutions. One more constraint must be given in order to obtain the unique solution, and these solvers cannot solve the intrinsic parameters, such as the focal length or principal point. Hence, some works of literature [20,21] proposed that the focal length could be estimated simultaneously as the pose estimation, and the minimum number of point correspondence sets is four. These methods solved the problem that the camera lens is unknown or the zoom lens is used. When the short focal lens or fisheye lens is used, image distortion exists. In this case, the distortion should be estimated to improve the accuracy. The literature from [22] proposed a method to estimate the pose, distortion and focal length simultaneously by using five 2D–3D point correspondences, called P5Pfr. However, it should be noted that the distortion here refers to the radial distortion—most of the distortion is radial distortion. Although the pose only contains six DOF (degrees of freedom), each degree of freedom contains trigonometric functions and is coupled to each other. If the minimum point set is used for estimation, the computational process is nonlinear and computational complexity is high. However, when the number of points used is not less than six, the pose estimation can be directly solved linearly [23,24,25], and the corresponding method is called DLT (direct linear transform). As the number of points used increases, the number of estimable parameters increases or the computational complexity decreases from a nonlinear to a linear solution. Of course, the difficulty of obtaining more accurate points also increases. Therefore, the choice of estimation method depends on the number of accurate points that can be obtained in the FOV (field of view) and whether the partial intrinsic parameters need to be estimated. In order to reduce complexity and improve precision, some parameters of the pose measured by the sensors can be used as prior knowledge. For example, the IMU (inertial measurement unit) is used to obtain a vertical direction [26,27,28,29], or RTK (real-time kinematic) is used to obtain camera positions [30,31,32]. These methods can reduce the number of required point correspondences while the number of estimated parameters is unchanged, and the accuracy and calculation speed are both improved.

Similar to the PnP problem, the minimum line set required to solve the PnL problem is also three, which is called the P3L (perspective-3-line) method [33,34]. The difference is that these methods have up to eight solutions, the computational complexity is higher, and the accuracy and computational speed are both decreased. More parameters can be estimated by using more 2D–3D line correspondences, reducing the computational complexity. When the number of 2D–3D line correspondences used is not less than six, it can be directly solved linearly [35]. Simultaneously, the computational complexity changes from the nonlinear solution of the P3L methods to a linear solution, which can improve the computational speed and accuracy. In addition, some methods use sensors to measure the partial pose information in advance, such as vertical direction [36,37] or camera position [38], to reduce the number of 2D–3D line correspondences required and improve the accuracy and computational speed. Furthermore, without reducing the number of 2D–3D line correspondences, some intrinsic parameters, such as focal length, can be simultaneously estimated.

There is a category of methods between the point-based and line-based correspondences, such as the pose estimation methods using vanishing points [39,40]. These methods use 2D vanishing point-3D parallel line correspondences, and our proposed method in this paper belongs to this category. One vanishing point can provide two constraints; hence, when there is no other prior knowledge, at least three vanishing points are needed to estimate the pose, and at least four vanishing points are needed if the focal length is estimated simultaneously. Grammatikopoulos [41] used two vanishing points to estimate the camera pose when two parallel line sets are perpendicular to each other and when the origin of the world frame is the intersection. Guo [42] used a single vanishing point to complete this job, but the disadvantage is that one orientation needs to be measured in advance.

In this paper, we propose a new method for estimating the focal length and pose by using two vanishing points and the camera’s position. In the camera frame, the camera’s position and each vanishing point can determine two lines. Additionally, according to the definition of vanishing point, the angle between the two lines, which is a function of the focal length, is equal to the angle between the corresponding two parallel line sets, which are known in the world frame. Hence, an equation can be obtained with an unknown parameter, i.e., the focal length. Then, the focal length is estimated efficiently. In addition, the vanishing point can determine the unit direction vector of the corresponding parallel lines in the camera frame after the focal length estimation. Because the unit direction vector in the world frame is the input, it is known. Then, the transformation between the two unit direction vectors in the camera frame and the world frame, respectively, can be obtained using the rotation matrix between the camera frame and the world frame. There are two vanishing points; hence, two transformations can be obtained. The transformation of the unit direction vectors can be regarded as the transformation of 3D points; that is, a 3D point in a camera frame, whose coordinates are equal to the values of the corresponding unit direction vector in the camera frame, can be transformed to a 3D point in a world frame whose origin is located at the origin of the camera frame, whose coordinates are equal to the values of the corresponding unit direction vector in the world frame. This is the key point to estimate the pose in this paper. The experimental results show our proposed method performs well in terms of numerical stability, noise sensitivity and computational speed in synthetic data and real images.

The remainder of the paper is organized as follows. In Section 2, we provide the derivation of the focal length and pose estimation; Section 3 provides the experiments and results to show how well our method performs; Section 4 and Section 5 are the discussion and conclusions, respectively.

## 2. Proposed Method

**Figure 1:**
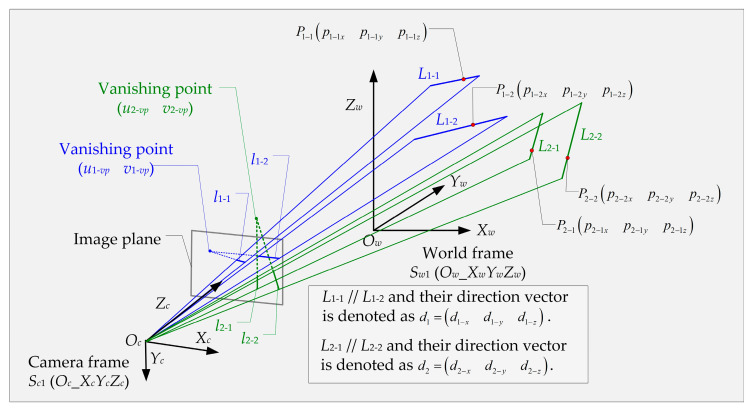
The geometric construction of this paper includes two sets of parallel lines (blue and green), the corresponding projections and vanishing points on the image plane.

This paper provides two sets *L_i_* (*i* = 1,2) of parallel lines with known direction vectors and a camera position *O_c_* in the world frame *S_w_*_1_ (*O_w__X_w_Y_w_Z_w_*) to estimate the pose and focal length. To simplify the derivation, we assume each set has two 3D lines, namely *L_i−j_* (*j* = 1,2), and then the corresponding projections of them on the image plane are denoted as *l_i−j_*. The geometric construction is illustrated in Figure 1.

Next, we will use two steps to estimate the pose and focal length, respectively.

### 2.1. Focal Length Estimation

In the camera frame *S_c_*_1_ (*O_c__X_c_Y_c_Z_c_*), the unit direction vector of the 3D line *L_i−j_* is denoted as $d_{i}=\left(\begin{array}{ccc} d_{i-x} & d_{i-y} & d_{i-z} \end{array}\right)$, which is unknown. Additionally, a 3D point $P_{i-j}\left(\begin{array}{ccc} p_{i-jx} & p_{i-jy} & p_{i-jz} \end{array}\right)$, which is also unknown, is on the 3D line *L_i−j_*. Now, the line *L_i−j_* can be written as

(1) $$L_{i-j}=P_{i-j}+k_{i-j}\cdot d_{i}$$

**Figure 2:**
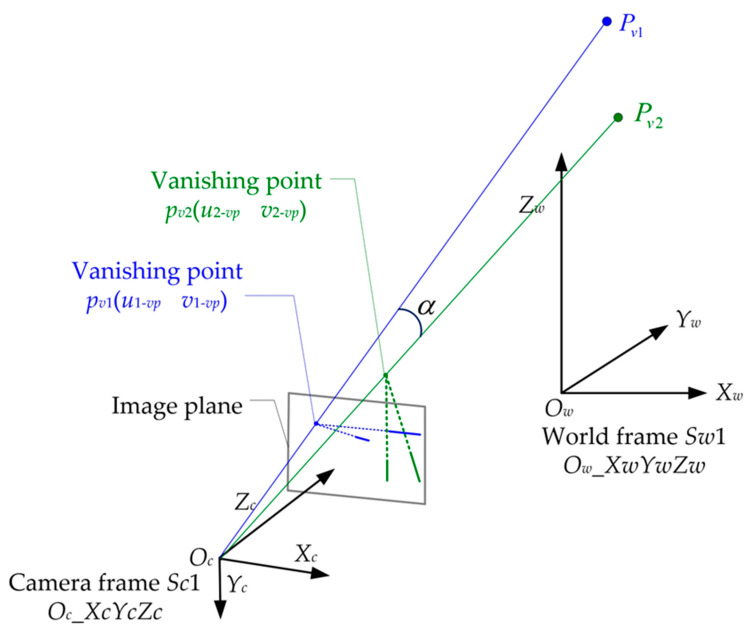
Two vanishing points were used for focal length estimation.

Here, *k_i−j_* is an arbitrary scale factor. The vanishing point on the image plane is the projection of the 3D point located at the infinity spatial place. Here, we assume these 3D points can be seen and denoted as $P_{v1},P_{v2}$ in this paper. According to Equation (1), their coordinates can be written as

(2) $$\begin{array}{l} P_{v1}=k_{v1}\cdot d_{1}\;,\;\;\;\;\;\;k_{v1}\to \infty \\ P_{v2}=k_{v2}\cdot d_{2},\;\;\;\;\;\;k_{v2}\to \infty \end{array}$$

where $k_{v1},k_{v2}$ are the scale factors of the 3D points located at the infinity spatial place. Their projections on the image plane are denoted as $p_{v1}\left(\begin{array}{cc} u_{1-vp} & v_{1-vp} \end{array}\right),p_{v2}\left(\begin{array}{cc} u_{2-vp} & v_{2-vp} \end{array}\right)$. The geometric construction is illustrated in Figure 2.

In Figure 2, *α* is not only the angle between the lines $O_{c}P_{v1}$ and $O_{c}P_{v2}$ in the world frame *S_w_*_1_ but is also the angle between the lines $O_{c}p_{v1}$ and $O_{c}p_{v2}$ in the camera frame *S_c_*_1_. The expressions of the unit direction vectors of the lines $O_{c}P_{v1}$ and $O_{c}P_{v2}$ in the world frame *S_w_*_1_ can be written as

(3) $$\begin{array}{l} d_{v1}=\underset{k_{v1}\to \infty }{\lim}\frac{k_{v1}d_{1}-O_{c}}{\left\|k_{v1}d_{1}-O_{c}\right\|}=d_{1}\\ d_{v2}=\underset{k_{v2}\to \infty }{\lim}\frac{k_{v2}d_{2}-O_{c}}{\left\|k_{v2}d_{2}-O_{c}\right\|}=d_{2} \end{array}$$

Then, we can calculate the angle *α* using

(4) $$\cos\alpha =\frac{d_{v1}\cdot d_{v2}}{\left\|d_{v1}\right\|\cdot \left\|d_{v2}\right\|}$$

In the camera frame *S_c_*_1_, the expressions of the direction vectors of the lines $O_{c}p_{v1}$ and $O_{c}p_{v2}$ can be written as

(5) $$\begin{array}{l} O_{c}p_{v1}=\left(\begin{array}{ccc} u_{1-vp} & v_{1-vp} & f \end{array}\right)\\ O_{c}p_{v2}=\left(\begin{array}{ccc} u_{2-vp} & v_{2-vp} & f \end{array}\right) \end{array}$$

Here, *f* is the focal length in pixels. According to the characteristic of angle *α*, we can obtain

(6) $$\cos\alpha =\frac{O_{c}p_{v1}\cdot O_{c}p_{v2}}{\left\|O_{c}p_{v1}\right\|\cdot \left\|O_{c}p_{v2}\right\|}=\frac{u_{1-vp}\cdot u_{2-vp}+v_{1-vp}\cdot v_{2-vp}+f^{2}}{\sqrt{u_{1-vp}^{2}+v_{1-vp}^{2}+f^{2}}\cdot \sqrt{u_{2-vp}^{2}+v_{2-vp}^{2}+f^{2}}}$$

Let $\cos\alpha =m_{1}$, $u_{1-vp}\cdot u_{2-vp}+v_{1-vp}\cdot v_{2-vp}=m_{2}$, $u_{1-vp}^{2}+v_{1-vp}^{2}=m_{3}$ and $u_{2-vp}^{2}+v_{2-vp}^{2}=m_{4}$. Then, we can simplify Equation (6) as

(7) $$\left(m_{1}^{2}-1\right)\cdot f^{4}+\left(m_{1}^{2}m_{3}+m_{1}^{2}m_{4}-2m_{2}\right)\cdot f^{2}+m_{1}^{2}m_{3}m_{4}-m_{2}^{2}=0$$

Here, $f^{2}$ is regarded as the unknown parameter, and the equation is a quadratic equation with one unknown. Two solutions of $f^{2}$ exist. Since f>0 and $f^{2}>0$, we can obtain a unique solution.

### 2.2. Pose Estimation

Using the standard pinhole camera model, we can obtain the projection *l_i−j_* $\left(\begin{array}{cc} u_{i-j} & v_{i-j} \end{array}\right)$ of the line *L_i−j_* as follows.

(8) $$\begin{array}{l} u_{i-j}=f\frac{p_{i-jx}+k_{i-j}\cdot d_{i-x}}{p_{i-jz}+k_{i-j}\cdot d_{i-z}}\\ v_{i-j}=f\frac{p_{i-jy}+k_{i-j}\cdot d_{i-y}}{p_{i-jz}+k_{i-j}\cdot d_{i-z}} \end{array}$$

Here, *f* is the focal length. If *k_i−j_* goes to infinity and *d_i-z_* is not zero, the projection is the vanishing point and can be written as

(9) $$\begin{array}{l} u_{i-vp}=\underset{k_{i-j}\to \infty }{\lim}f\frac{p_{i-jx}+k_{i-j}\cdot d_{i-x}}{p_{i-jz}+k_{i-j}\cdot d_{i-z}}=f\frac{d_{i-x}}{d_{i-z}}\\ v_{i-vp}=\underset{k_{i-j}\to \infty }{\lim}f\frac{p_{i-jy}+k_{i-j}\cdot d_{i-y}}{p_{i-jz}+k_{i-j}\cdot d_{i-z}}=f\frac{d_{i-y}}{d_{i-z}} \end{array}$$

It can be seen that the vanishing point is decided only by the direction vector of the corresponding parallel lines in the camera frame.

Through feature extraction, we can obtain the expression of the line *l_i−j_*. Actually, two expressions can be obtained for each set of parallel lines. Then, we can calculate the position of the vanishing point on the image plane using the two expressions. That means $\left(\begin{array}{cc} u_{i-vp} & v_{i-vp} \end{array}\right)$ is known. Consequently, according to Equation (9), the direction vector of the corresponding 3D lines in the camera frame can be given using

(10) $$d_{i}=d_{i-z}\left(\begin{array}{ccc} \frac{u_{i-vp}}{f} & \frac{v_{i-vp}}{f} & 1 \end{array}\right)$$

Then, the corresponding unit direction vector in the camera frame can be written as

(11) $$d_{i-c}=\frac{1}{\sqrt{u_{i-vp}^{2}+v_{i-vp}^{2}+f^{2}}}\left(\begin{array}{ccc} u_{i-vp} & v_{i-vp} & f \end{array}\right)$$

It can be seen that the unit direction vector of the parallel lines in the camera frame can be determined by the corresponding vanishing point. Since the vanishing point can be calculated, the unit direction vector $d_{i-c}$ of the parallel lines in the camera frame *S_c_*_1_ is known. In addition, as the input, the unit direction vector $d_{i-w}$ of the parallel lines in the world frame *S_w_*_1_ is also known. According to the rigid body transformation, an Equation can be given as follows.

(12) $$d_{i-c}=R_{w-c}\cdot d_{i-w}$$

Here, $R_{w-c}$ is the rotation matrix between the world frame *S_w_*_1_ and camera frame *S_c_*_1_, which is unknown and contains all the parameters of pose that we require for the estimate in this paper. This equation is similar to the traditional frame transformation that is written as

(13) $$P_{c}=R_{w-c}\cdot P_{w}+t$$

The meaning of Equation (13) is that a 3D point *P_w_* in the world frame *S_w_*_1_ can be transformed to *P_c_* in the camera frame *S_c_*_1_ through the rotation matrix $R_{w-c}$ and the translation vector *t*. If we let *t* = 0, the world frame and camera frame have the same origin, and then we can assume

(14) $$\begin{array}{l} P_{c}=d_{i-c}\\ P_{w}=d_{i-w} \end{array}$$

**Figure 3:**
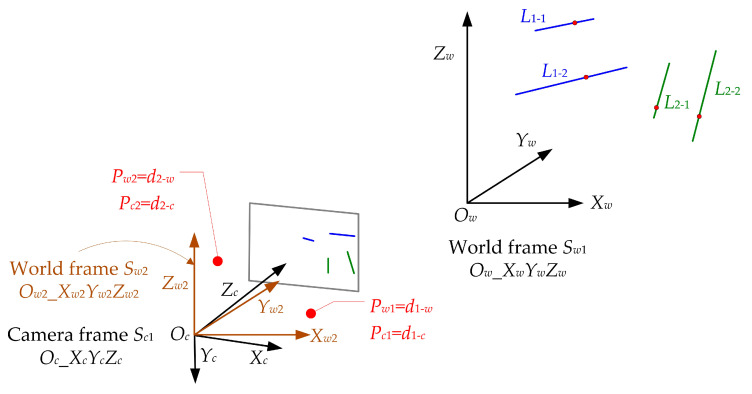
New world frame (brown) and two virtual 3D points (red).

Now, Equations (12) and (13) are the same. Then, we can say that Equation (12) is the transformation for the 3D point when the translation vector is zero, and the coordinate of the 3D point is equal to the value of the unit direction vector. Note that this is the paper’s key point for estimating the camera pose. In detail, here we regard the transformation between the unit direction vectors as the transformation between the 3D points is a PnP problem when the translation vector is zero. To obtain the case where the translation vector is zero, we must establish a new world frame and two virtual 3D points, as shown in Figure 3.

A new world frame, *S_w_*_2_ (*O_w_*_2__*X_w_*_2_*Y_w_*_2_*Z_w_*_2_), is established in Figure 3. It is parallel to the original world frame S*_w_*_1,_ and only translation exists between the two world frames. When the origin of the world frame *S_w_*_2_ is located at the camera position *O_c_*, we can obtain the transformation between the two world frames as follows.

(15) $$S_{w2}=S_{w1}-O_{c}$$

In addition, according to the unit direction vectors, we established two virtual spatial points plotted in red in Figure 3. Their coordinates, both in the camera frame *S_c_*_1_ and world frame *S_w_*_2_, are also shown in Figure 3, and then their transformation can be written as

(16) $$P_{ci}=R_{w-c}\cdot P_{wi}$$

**Figure 4:**
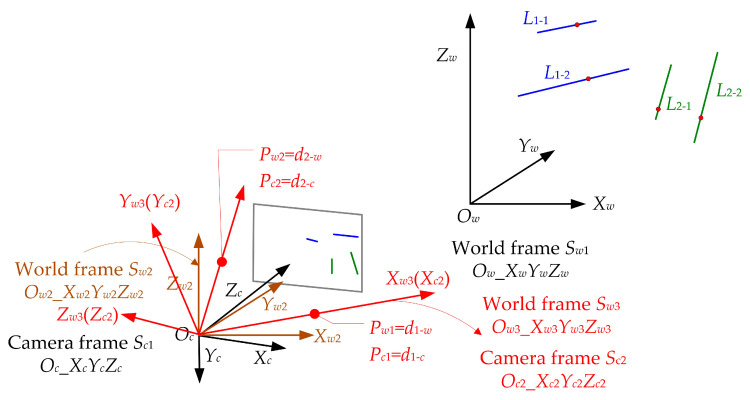
New camera frame *S_c_*_2_ and new world frame *S_w_*_3_ are plotted in red.

Here, $R_{w-c}$ is both the rotation matrix between world frame *S_w_*_2_ and camera frame *S_c_*_1_ and the rotation matrix between world frame *S_w_*_1_ and camera frame *S_c_*_1_. Next, the two virtual spatial points will be used to estimate the rotation matrix that contains all the information for the camera pose. Before that, two intermediate frames need to be established, i.e., a new world frame, *S_w_*_3_ (*O_w_*_3__*X_w_*_3_*Y_w_*_3_*Z_w_*_3_), and a new camera frame, *S_c_*_2_ (*O_c_*_2__*X_c_*_2_*Y_c_*_2_*Z_c_*_2_). The two frames coincide in space, and their origin is located at the camera position *O_c_*, as shown in Figure 4.

Each axis of the new camera frame *S_c_*_2_ can be calculated using

(17) $$\begin{array}{l} \vec{O_{c2}X_{c2}}=\frac{\vec{O_{c}P_{c1}}}{\left\|\vec{O_{c}P_{c1}}\right\|}\\ \vec{O_{c2}Z_{c2}}=\frac{\vec{O_{c2}X_{c2}}\times \vec{O_{c}P_{c2}}}{\left\|\vec{O_{c2}X_{c2}}\times \vec{O_{c}P_{c2}}\right\|}\\ \vec{O_{c2}Y_{c2}}=\vec{O_{c2}Z_{c2}}\times \vec{O_{c2}X_{c2}} \end{array}$$

Then, the camera frame *S_c_*_2_ can be transformed into the camera frame *S_c_*_1_ using

(18) $$\begin{array}{l} S_{c2}=T_{c\_c2}\cdot S_{c1}\\ T_{c\_c2}={\left[\begin{array}{ccc} \vec{O_{c2}X_{c2}} & \vec{O_{c2}Y_{c2}} & \vec{O_{c2}Z_{c2}} \end{array}\right]}^{T} \end{array}$$

Each axis of the new world frame *S_w_*_3_ can be calculated using

(19) $$\begin{array}{l} \vec{O_{w3}X_{w3}}=\frac{\vec{O_{c}P_{w1}}}{\left\|\vec{O_{c}P_{w1}}\right\|}\\ \vec{O_{w3}Z_{w3}}=\frac{\vec{O_{w3}X_{w3}}\times \vec{O_{c}P_{w2}}}{\left\|\vec{O_{w3}X_{w3}}\times \vec{O_{c}P_{w2}}\right\|}\\ \vec{O_{w3}Y_{w3}}=\vec{O_{w3}Z_{w3}}\times \vec{O_{w3}X_{w3}} \end{array}$$

Then, the world frame *S_w_*_3_ can be transformed into the world frame *S_w_*_2_ using

(20) $$\begin{array}{l} S_{w3}=T_{w2\_w3}\cdot S_{w2}\\ T_{w2\_w3}={\left[\begin{array}{ccc} \vec{O_{w3}X_{w3}} & \vec{O_{w3}Y_{w3}} & \vec{O_{w3}Z_{w3}} \end{array}\right]}^{T} \end{array}$$

**Figure 5:**
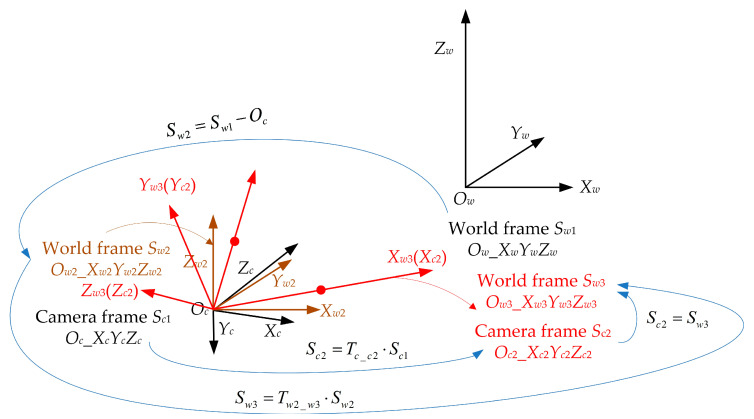
Transformations between different frames.

Now, we have obtained the transformations between different frames, as shown in Figure 5.

According to the known transformations between different frames, the pose estimation, that is, the transformation from world frame *S_w_*_1_ to camera frame *S_c_*_1_, can be given using

(21) $$\begin{array}{l} S_{c1}=T_{w\_c}\cdot S_{w1}+t_{w\_c}\\ T_{w\_c}=T_{c\_c2}^{-1}\cdot T_{w2\_w3}\\ t_{w\_c}=-T_{c\_c2}^{-1}\cdot T_{w2\_w3}\cdot O_{c} \end{array}$$

Now, the pose estimation is finished. Note that the solving process is similar to the method proposed in [38] but has an essential difference, which will be discussed in Section 4.

## 3. Experiments and Results

In this Section, first, we will thoroughly and directly test our proposed method with synthetic mass data, including numerical stability, noise sensitivity and computational speed of both pose and the focal length estimation. Simultaneously, the performance of our proposed method will be compared with that of some other existing SOTA (state-of-the-art) solvers (i.e., P3P [16], P3L [11], GPnPf (the Gauss–Newton method for the perspective-n-point and focal length) [20], RPnP (the robust O (n) solution to the perspective-n-point) [6], and DLT [23]), which involves a nonlinear algorithm, linear algorithm, point-based algorithm and line-based algorithm. In addition, some can only estimate the pose, and some can estimate both the pose and the focal length.

Second, the prior knowledge (i.e., camera position) used in our proposed method cannot be absolutely correct, which may affect the accuracy of the pose and focal length estimation, seriously or not. Hence, the robustness of our proposed method of camera position noise needs to be tested.

Last, we indirectly evaluate the performance of our proposed method with real images and compare it with the SOTA solvers to show if it can work well with real scenarios or not.

### 3.1. Synthetic Data

Here, synthetic mass data is generated by a virtual perspective camera with a standard pinhole camera model, whose resolution is 1280 × 800, the principal point is the center of the image, and the pixel size is 14 μm. In order to simplify the experiments, no distortion was added to the image, and this is reasonable in many cases where a short lens and fisheye lens are not used. Another reason is that the manufacturing and installation of the lens are both accurate, which means the distortion is small. The camera is located at [2, 2, 2] in meters in the world frame, and the focal length is 50 mm.

For the P3P, GPnPf, RPnP and DLT solvers, 2D–3D point correspondences are needed. For the P3L solver, 2D–3D line correspondences are needed, and for our proposed method, 2D vanishing point-3D parallel line correspondences are needed. Hence, random 3D points, lines and parallel lines are generated in a box of [−17 17] × [−11 11] × [50 60] in meters in the camera frame. Then, the 2D correspondences are generated through the virtual perspective camera. The numbers of the 2D–3D correspondences are all three thousand for all the methods in this paper. Now, the synthetic data is generated and contains three thousand 2D–3D point correspondences, three thousand 2D–3D line correspondences, and three thousand 2D vanishing point-3D parallel line correspondences.

In this section, according to the minimal set of 2D–3D correspondences for each method, three 2D–3D point correspondences, four 2D–3D point correspondences, five 2D–3D point correspondences, six 2D–3D point correspondences, three 2D–3D line correspondences and two 2D vanishing point-3D line correspondences are randomly selected from the synthetic data for P3P, GPnPf, RPnP, DLT, and P3L and our proposed method, respectively, for each trial.

#### 3.1.1. Robustness to Camera Position Noise

The camera position can be measured by equipment mounted on a camera, such as the IMU (inertial measurement unit) and RTK (real-time kinematic), or by other tools, such as the total station. They have high positioning accuracy, better than 3 cm [19]. In this section, we want to know how the camera position noise affects the accuracy of our proposed method because the camera position is prior knowledge, which differs from other methods. Hence, we solely analyze the robustness of the camera position noise for our proposed method.

**Figure 6:**
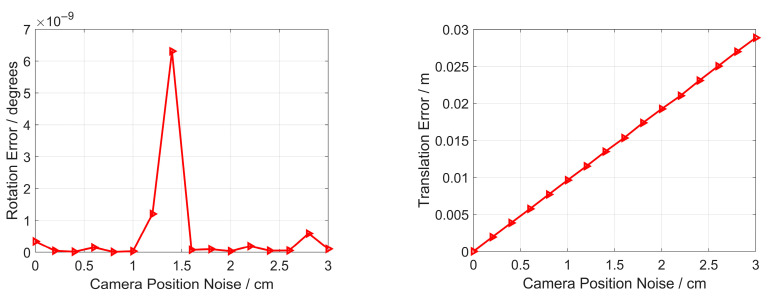

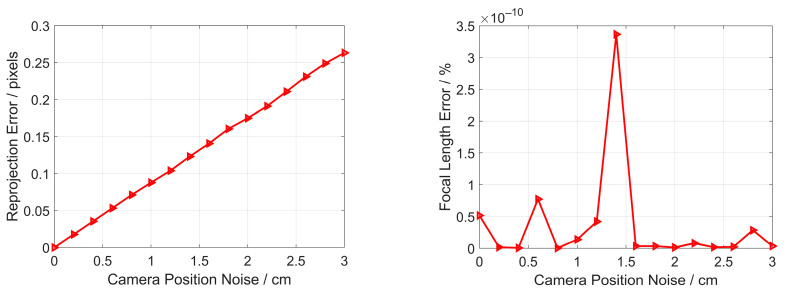
Robustness to camera position noise for the rotation error (**Top left**), translation error (**Top right**), reprojection error (**Bottom left**) and focal length error (**Bottom right**).

Here, Gaussian noise, whose deviation level varies from 0 to 3 cm, is added to the camera position. For each noise level, 10,000 random trials are independently performed. Then, the mean errors of rotation, translation, reprojection and focal length are reported in Figure 6.

In Figure 6, the rotation error and focal length error are both low, which can be regarded as zero, even though error spikes exist. The reason is that the rotation and focal length estimation do not involve the camera position, which can be explained with Equations (6) and (21). In addition, as the camera position noise increases, so do the translation and reprojection errors. The reason is that the translation estimation involves the camera position, which can be explained with Equation (21). For reprojection, it is related to rotation, focal length and translation; hence, it is affected by the camera’s position. When the camera position noise is 3 cm, the translation and reprojection errors both reach the maximums, which are 0.028 m and 0.26 pixels, respectively. The errors are both small and show that our proposed method has strong robustness to the camera position noise.

#### 3.1.2. Numerical Stability

**Figure 7:**
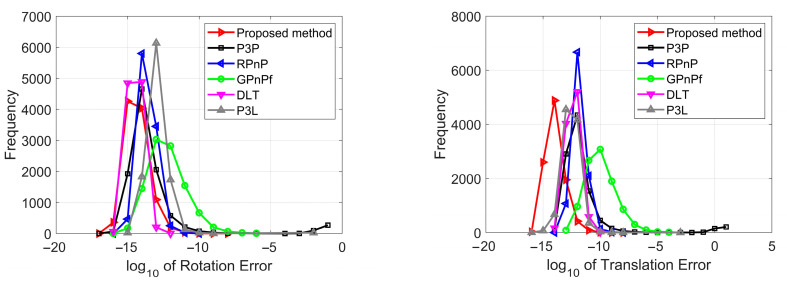

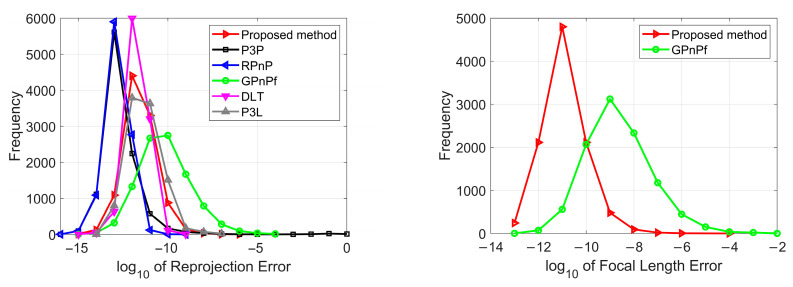
Numerical stability for our proposed method (red), P3P (black), RPnP (blue), GPnPf (green), DLT (purple) and P3L (gray). The (**top left**) is the rotation error, the (**top right**) is the translation error, the (**bottom left**) is the reprojection error, and the (**bottom right**) is the focal length error.

We tested our proposed method in terms of numerical stability in this section. A total of 10,000 trials were performed independently using synthetic data with no noise added. The performance of the rotation, translation and projection estimation was compared to the other five methods, and the performance of the focal length estimation was compared only to the GPnPf method because the other four methods could not estimate the focal length. The results of numerical stability are reported in Figure 7.

Figure 7 shows the distribution of the rotation, translation, projection and focal length error, and all six methods have good numerical stability. To be specific, the DLT method has the best performance, and our proposed method has the second in terms of rotation error; our proposed method has the best performance, and the P3L method has the second in terms of translation error; the RPnP method has the best performance, and our proposed method has the fourth in terms of reprojection error; our proposed method has the best performance, and the GPnPf method has the second in terms of focal length error. As a whole, our proposed method has the best performance in terms of numerical stability.

#### 3.1.3. Noise Sensitivity

**Figure 8:**
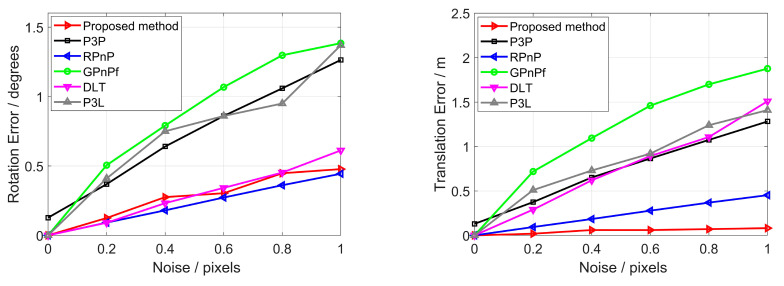

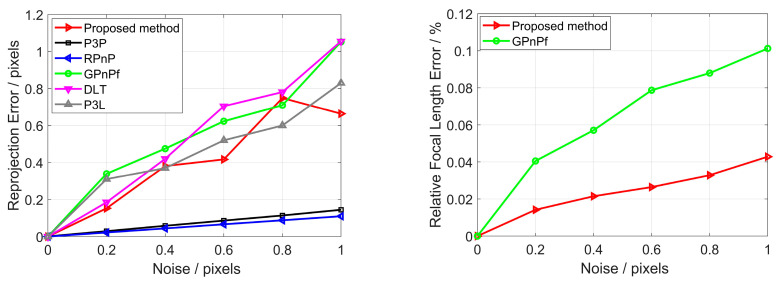
Noise sensitivity for our proposed method (red), P3P (black), RPnP (blue), GPnPf (green), DLT (purple) and P3L (gray). The (**top left**) is the rotation error, the (**top right**) is the translation error, the (**bottom left**) is the reprojection error, and the (**bottom right**) is the focal length error.

We tested our proposed method in terms of noise sensitivity in this section. The trials were performed independently using synthetic data with noise added. Noise may exist in the 2D feature or 3D feature. Because the 3D feature will be transformed into a 2D feature, 2D noise can reflect the 3D noise. Hence, we only added zero-mean Gaussian noise onto the 2D points and lines, and the noise deviation level varies from 0 to 1 pixel. A total of 10,000 trials were performed independently for each method, respectively, and the performance of the rotation, translation and projection estimation was compared to the other five methods. The performance of the focal length estimation was compared only to the GPnPf method because the other four methods could not estimate the focal length. The results of noise sensitivity are reported in Figure 8.

From Figure 8, it can be seen that as the noise increases, so does the rotation error, the translation error, the reprojection error and the focal length error. To be specific, the RPnP, DLT and our proposed method have similar performances, and the RPnP method performs slightly better than our proposed method in terms of rotation error; our proposed method has the best performance, and the RPnP method has the second in terms of translation error, and they both perform much better than the other four methods. The RPnP and P3P methods have similar performances, and both perform better than the other four methods in terms of reprojection error. In addition, our proposed method has the third in terms of reprojection error. Our proposed method has the best performance, and the GPnPf method has the second in terms of focal length error, and our proposed method performs much better than the GPnPf method. As a whole, our proposed method has the best or second performance in terms of noise sensitivity.

#### 3.1.4. Computational Speed

In this section, 10,000 independent trials using synthetic data with no noise added were conducted on a 3.3 GHz two-core laptop for all six methods, respectively, to test the computational speed. Then, the mean computational times are reported in Table 1.

From Table 1, we can see that our proposed method has the best performance in terms of computational speed, and the DLT has the second. Specifically, our proposed method’s computational speed is 3.2 times, 3.8 times, 14.6 times, 1.5 times and 3.0 times that of the latter five methods, respectively. This shows that our proposed method has fast computational speed while having a good performance of numerical stability and noise sensitivity.

### 3.2. Real Images

In Section 3.1, we have shown that our proposed method can work well with synthetic data directly. To fully test our proposed method, we will now use real images to show whether it works well with real scenarios. The cameras were placed in real scenarios, but the ground truths of their poses are not known. This problem suggests that we cannot directly test our proposed method. Here, an indirect method was established to test it.

**Figure 9:**
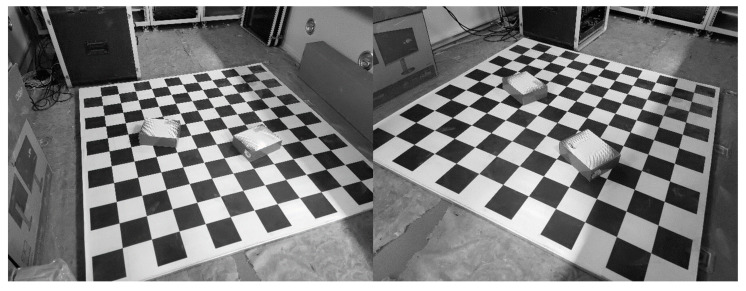
Real images from two different views.

First, many lines and points, whose positions are known as ground truth, were placed in the FOV, and then we chose some of them to estimate the pose and focal length for our proposed method and other SOTA methods. After estimating, stereo vision [43] was used to measure the 3D positions of the left points as the measured values. The accuracy of the measured value is affected by the camera’s pose and focal length. The pose and focal length were estimated by our proposed method and other SOTA methods. Hence, the measurement accuracy between the measured value and ground truth can reflect the accuracy of our proposed method. Next, we set up the real scenarios and captured real images from two different views by the cameras [38], shown in Figure 9.

**Figure 10:**
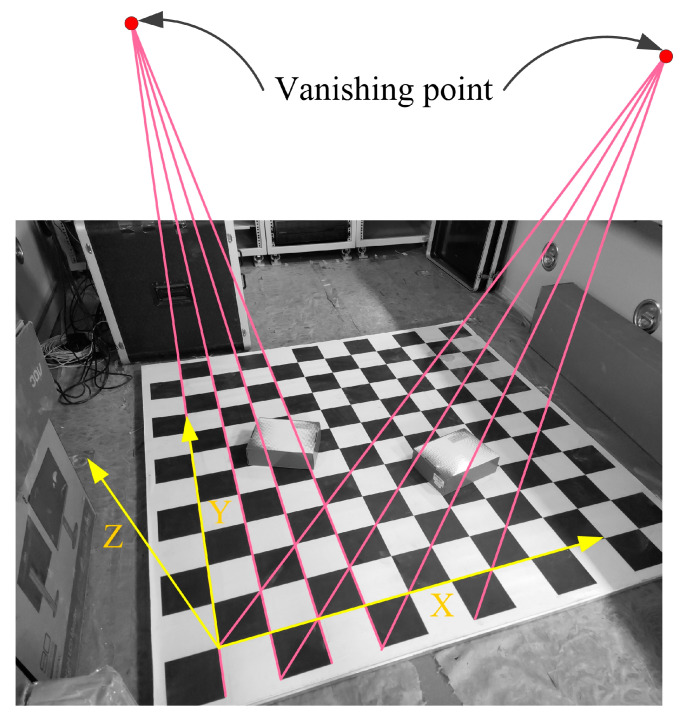
Two vanishing points were extracted for the focal length and pose estimation.

The checkerboard was placed in the FOV, and the size was known. Hence, there are many sets of parallel lines, and their unit direction vectors are known. We chose two sets for our proposed method to estimate the focal length and pose, as shown in Figure 10.

The world frame (yellow) was established, as shown in Figure 10, and two vanishing points were obtained from the two sets of parallel lines (red). In addition, for the P3P, GPnPf, RPnP, DLT and P3L solvers, three points, four points, five points, six points and three lines from the checkerboards were randomly chosen to estimate the focal length and pose. The camera positions were measured by a total station for our proposed method. After the focal length and pose estimations, the stereo vision was used to measure the positions of the left points on the checkerboards as measured values. Then, we obtained the mean relative position errors between the measured values and ground truths, as shown in Table 2. Moreover, the reprojections of the left points can then be obtained, and the mean reprojection errors between the reprojection and projection are also reported in Table 2.

Our proposed method and P3P have the best performance in terms of the mean relative position error; our proposed method has the third performance, and RPnP has the first in terms of the mean reprojection error. As a whole, our proposed method performs best in real images.

In addition, we obtained the computational time of all the methods and our proposed method has the best performance. Specifically, the computational speed of our proposed method is 2.5 times, 3.4 times, 14.2 times, 1.6 times and 3.1 times that of the latter five methods, respectively. This is basically consistent with the results in the synthetic data.

For real images, there are many factors here that affect our proposed method, such as noise and error in the camera’s position. In fact, we took these factors into account when setting up our real scenario in this section. When extracting the feature points, we used the sub-pixel extraction algorithm, which will introduce noise. This is also related to the imaging quality, so the noise cannot be quantified. In addition, in the experiment, we used the total station to obtain the camera position because the total station itself has an error; hence, the error of the camera position is also introduced in the real scenario. It is clear that we should consider the influence of these factors when analyzing the performance of our proposed method for the real scenario. Under the influence of these factors, our method still shows good performance. In addition, due to the real scenario limitations, it is difficult to set up many real scenarios. Therefore, we adopted synthetic data to simulate different scenarios in Section 3.1. Using a combination of a small number of real scenarios and a large number of synthetic scenarios, and in the case of introducing various types of errors, shows our proposed method has good performance.

## 4. Discussion

This paper uses two vanishing points and a camera’s position to estimate the focal length and pose simultaneously. To our best knowledge, this is the first paper to perform this job using vanishing points and a camera’s position. Using the camera position as the prior knowledge can simplify the estimation problem and improve accuracy and efficiency. Unlike other existing methods, our proposed method does not involve nonlinear computation and multi-solution phenomenon and needs only two vanishing points. In computer vision, our proposed method can estimate the camera pose more quickly in the case of multiple vanishing points. It is another idea to be used to estimate the pose, which is complementary to other calibration methods. The differences and advantages of the proposed method and future work will be discussed as follows.

### 4.1. Differences and Advantages

The first difference is that our proposed method uses the camera position as the prior knowledge, and it can simplify the problem. In fact, many existing methods also use some prior knowledge and also simplify the problem. However, the costs of using prior knowledge for different methods are different. Additionally, the corresponding benefits are different. Some methods require expensive equipment with large sizes and precision mechanical structures to obtain prior knowledge and might not achieve a very good effect. However, the camera’s position can be given by RTK, which is cheap and has a small size. In addition, it has strong robustness (Section 3.1.1) to the camera’s position and good performance in terms of numerical stability, noise sensitivity and computational speed (Section 3.1.2, Section 3.1.3 and Section 3.1.4). This means we can obtain good benefits at a low cost. The advantage can also be seen indirectly in Section 3.2. Last, the rotation and focal length estimation do not involve the camera position; hence, they have no error when the camera noise exists (Section 3.1.1).

The second difference is that our proposed method does not involve nonlinear iterations. Some existing methods, e.g., P3P, RPnP and GPnPf, need to solve the nonlinear equation, and in order to avoid the optimal local solution, iteration is needed. Although nonlinear iteration could improve the accuracy, the computational speed is decreased, as shown in Section 3.1.4. This is the main reason why our proposed method and DLT have the best performance in terms of computational speed. In addition, improving the accuracy does not mean we can always obtain the optimal global solution, and this leads to our proposed method perhaps having higher accuracy, as shown in Section 3.1.3.

The third difference is that our proposed method has no multi-solution phenomenon. When we estimate the focal length, a quadratic equation with one unknown must be solved. When we estimate the pose, the computational process mainly involves multiplication and matrix operations. Hence, there is no multi-solution phenomenon. Because one more constraint is needed to disambiguate the multi-solution phenomenon, the computational speed will decrease, and this is another reason why our proposed method has the best performance in terms of computational speed.

Last, the calculating process is similar to another method that we proposed in [38]; however, they have essential differences. The method in [38] needs to establish two planes in the world frame and camera frame and obtain their normal unit vectors. However, this paper directly uses the unit direction vectors of lines and does not establish the planes. Additionally, when we estimate the focal length, this paper uses the angle between two lines, not the two planes in [38]. This paper only requires the unit direction vectors of the 3D lines but does not need the positions in the space. The method in [38] needs both. It can be seen (although the forms of the equations and calculating process are similar) that the meanings of the two methods are totally different.

The main disadvantage is that our proposed method does not perform best in terms of reprojection error. The reason is that some other methods, such as P3P and RPnP, use iteration to refine the solution, and the corresponding cost function is to make the reprojection error minimal. Our proposed method has no refining process, which leads to the main disadvantage.

Briefly, our proposed method has the following advantages. (1) Only two vanishing points are needed; (2) it has no multi-solution phenomenon; (3) it has strong robustness to camera noise; (4) as a whole, it performs well in terms of numerical stability and noise sensitivity; (5) the computational speed is fast. The main disadvantage is that it does not perform best in terms of reprojection error.

### 4.2. Future Work

As described in Section 4.1, our proposed method has a main disadvantage for reprojection error. Hence, the main work in the future is to establish the cost function to minimize the reprojection error and simultaneously refine the solution. Another work will use other sensors to obtain additional prior knowledge, such as the IMUs, which can give two orientations of the camera. It also can simplify the problem. Additionally, it may be possible that the camera’s position and partial orientation are both used as prior knowledge to improve accuracy or estimate more intrinsic parameters.

## 5. Conclusions

This paper proposed a new method to estimate the focal length and pose based on two vanishing points and a camera’s position. The key point is to convert the transformation between the unit direction vectors to the transformation between the 3D points without translation. The experimental results show that, as a whole, our proposed method performs better than some existing state-of-the-art methods.

## Figures and Tables

**Table 1:** Computational time.

Method	Our Proposed Method	P3P	RPnP	GP4Pf	DLT	P3L
Computational time	0.43 ms	1.37 ms	1.64 ms	6.26 ms	0.65 ms	1.31 ms

**Table 2:** Mean relative position errors and mean reprojection errors.

Method	Proposed Method	P3P	RPnP	GPnPf	DLT	P3L
Mean relative error %	0.45	0.54	1.81	1.17	0.59	0.72
Mean reprojection/pixel	0.61	0.56	0.49	0.79	0.72	0.67

## Data Availability

The data presented in this study are available in the manuscript.
